# Supplementary material for: Chromothripsis during telomere crisis is independent of NHEJ, and consistent with a replicative origin
Source: Genome Res. 2019 May;29(5):737–49. doi: 10.1101/gr.240705.118 (PMC6499312; doi:10.1101/gr.240705.118)
Supplement: Supplemental Material [file supp_gr.240705.118_Supplemental_file_1.zip › contigs/annotated_contigs/DB73/contig.2.DB73_length_355_mean_cov_4.58309859155.docx]

**DB73_length_355_mean_cov_4.58309859155**

```
ATGTCTATTTATAGAACCCAGAAGGCCACCATGTCCTGGGCCAAGATACATACACTGCCACAAATACAGAATCAGCAGCAACACTCCAC
 >chrX:37543572-37543892 - E=8e-182
AGAGTATTTGTGTAATGTTTTACTTGACATCATGGTTAGCTTTAAAGGAAAAATTGGTAGCTTAGAATTATTAATCAGGACACTGAATA

TTTAGCATTAGATTTTATTTTAAGGCAAAGGATTATCACAGGTTTTACATTAAGTGTACTGTACATAAATGTATTATAAGGAGGGGCAA

TAAGCCAATAAATAACATCGCTTTAAACACTATAAAGTCCAGTTTATAGCG|AA|ATTGGAGGCTTGTCACAGTTGATATAACTATGTT
                                                    >chrX:33854806-33854843 - E=8e-08
G
```
